# Chiral Separation, Configuration Confirmation and Bioactivity Determination of the Stereoisomers of Hesperidin and Narirutin in *Citrus reticulata* Blanco

**DOI:** 10.3390/molecules28020873

**Published:** 2023-01-15

**Authors:** Bingtong Jiang, Sirong Cao, Jiayu Zhang, Zhaokun Wang

**Affiliations:** 1 School of Pharmacy, Binzhou Medical University, 346 Guanhai Road Laishan District, Yantai 264003, China; 2 College of Pharmaceutical Science, Yunnan University of Chinese Medicine, No. 1076 Yuhua Road, Chenggong District, Kunming 650500, China

**Keywords:** flavanone glycoside, citrus peel, chiral separation, epimer composition, anti-inflammatory activity

## Abstract

Hesperidin and narirutin are a class of flavanone glycosides, which are the main active constituents in *Citrus reticulata* Blanco. In the present study, a chiral HPLC-UV method with amylose tris(3,5-dimethylphenylcarbamate) as a stationary phase under a normal-phase mode was used to achieve the stereoselective separation of the C-2 diastereomers of hesperidin and narirutin simultaneously. The single epimer was then successfully prepared by applying semi-preparative chromatography, whose absolute configuration (*R*/*S*) was characterized by combining the experimental electronic circular dichroism (ECD) detection with time-dependent density functional theory (TDDFT) calculations. The epimer composition of these two chiral flavanone glycosides in *Citrus reticulata* Blanco was then determined, which was found to be slightly different in the herbs from different production regions. The anti-inflammatory activity of each prepared single epimer was further evaluated, and some differences between one pair of epimers of hesperidin and narirutin were observed, which suggested that the presence of different epimers should be considered in the quality evaluation and control of natural medicine.

## 1. Introduction

Flavanone components are a class of flavonoids, mostly in the form of glycosides, and no flavanone monomer compounds have been found in nature so far [1]. Flavanone glycosides are mainly found in citrus plants of the family of *Rutaceae*. For example, hesperidin, naringenin, narirutin, eriocitrin and neohesperidin are the most abundant components in the peel part of fruit, such as orange, grapefruit, sweet and lime, as well as lemon [2,3,4,5,6].

The substituents on the backbone structure of flavanone are diverse, including the methoxy, hydroxyl and isopentenyl groups, with biological activities such as anti-inflammatory [7], antioxidant [8], bactericidal [9], anti-cancer [10] and anti-virus [11] activities; therefore, these compounds have a great application value in the food and pharmaceutical fields. Due to the wide distribution and variety of biological activities, flavanone glycosides have already been chosen for the quality assessment and control of citrus herbal medicines, such as *Citri Reticulatae Pericarpium* (Chenpi) and *Citri Reticulatae Pericarpium Viride* (Qingpi) [12].

Unlike other flavonoids, flavanones have one special structural feature, with a chiral carbon at the C_2_ position in the basic skeleton structure (C_6_-C_3_-C_6_). Hence, flavanones consist of one pair of enantiomers. After the formation of flavanone 7-O-glycosides, these two enantiomers were transformed into one pair of epimers [13]. As we all know, the thalidomide tragedy served as a reminder that enantiomers have similar physicochemical properties but differ in their bioactivity. Significantly, the enantiomers of a chiral compound possess the exact same physicochemical properties in an achiral environment, while they (at least in principle) are characterized by different properties when interacting with a chiral entity. Thus, their biological activities sometimes differ. The same holds true for epimers. If one drug will be marketed as a racemate or mixture of stereoisomers, then the pharmacological activities of each single isomer must be documented to ensure the safety and efficacy of the drug.

Natural products are one of the essential resources in drug discovery. For natural active substances, the stereoisomerisms are exceedingly common, especially in flavonoids, alkaloids and triterpenes, which are generally regarded as the most effective materials in traditional Chinese medicine (TCM). Therefore, it is necessary to separate the stereoisomer and investigate the biological activity of each isomer.

*Citrus reticulata* Blanco (Rutaceae), an important cash crop, is mainly planted in the south of the Yangtze River in China [14]. As a common medicinal plant, *Citrus reticulata* Blanco has been proven to possess multiple functions, such as dredging meridian, promoting blood flow, delaying senescence, etc. [15,16]. Depending on the harvesting period, two different herbal medicines, *Citri Reticulatae Pericarpium* (Chenpi) and *Citri Reticulatae Pericarpium Viride* (Qingpi), can be obtained. A modern phytochemical study showed that *Citrus reticulata* Blanco is rich in flavonoid compounds. Among them, hesperidin and narirutin, as two flavanone components with high contents in the peels of *Citrus reticulata* Blanco [17], are two important active substances in Chenpi and Qingpi, and both of them are chiral compounds (Table 1).

Currently, several methods are used for the separation of hesperidin stereoisomers, such as high-performance liquid chromatography (HPLC) combined with chiral stationary phase (CSP) columns [18], supercritical fluid chromatography (SFC) [19] and microcapillary nano-upgrade liquid chromatography combined with the addition of a chiral selector into the mobile phase [20]. However, these methods are limited by their operational complexity, their high cost and their lack of an ability for the simultaneous quantification of multiple chiral flavonoids.

In the present study, we developed a highly efficient method for the simultaneous separation of the C-2 diastereomers of hesperidin and narirutin and the quantification of each epimer in Chenpi and Qingpi. Further, these single stereoisomers were obtained by semi-preparative liquid chromatography, whose stereo configurations were determined using electronic circular dichroism (ECD) data. In addition, the anti-inflammatory activity of each epimer was investigated in detail. In conclusion, the findings provide new insights for the study of chiral active ingredients in natural drugs and thus help in better understanding the potential mechanisms responsible for the clinical efficacy and safety of natural medicine.

## 2. Results and Discussion

### 2.1. Optimization of HPLC-UV Conditions

In order to obtain a simple, reliable, accurate and feasible method with the best separation effect, some chromatography parameters, including the types of chiral chromatographic columns, the compositions of the mobile phase as well as the column temperature on the separation of stereoisomers, were optimized.

**Figure 1:**
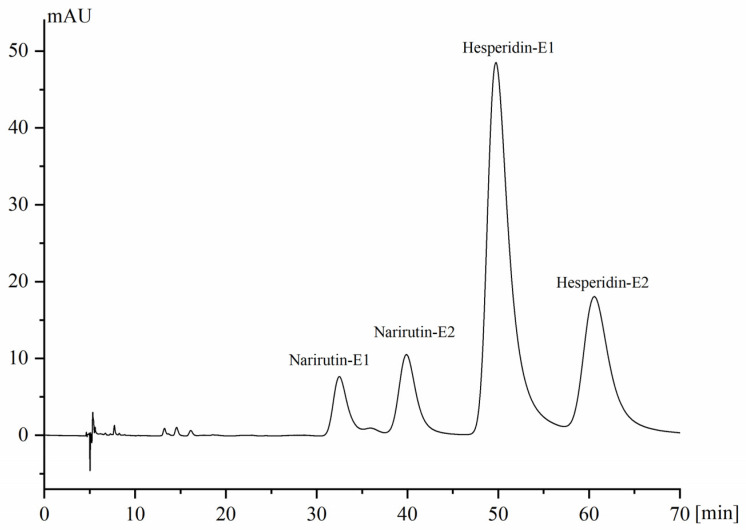
Simultaneous separation of the stereoisomers of hesperidin and narirutin in commercial standard substances under optimized conditions.

Based on the previous literature [21], the commercially available Chiral INC (cellulose tris(3,5-dichlorolphenylcarbamate)) and Chiral ND columns were tested for the chromatographic separation of hesperidin and narirutin epimers (Figure S1 and Table S1). In our research, it was found that the Chiral ND column showed the better resolution for both hesperidin and narirutin epimers, and it was used in our further study. The mobile phase, containing n-hexane, isopropanol/ethanol and formic acid in different proportions, was also investigated, which showed that a combination of n-hexane, isopropanol and formic acid at 62:38:0.25 (*v*/*v*/*v*) provided the best resolution for both hesperidin and narirutin epimers. Under the optimal mobile phase condition, the effects of column temperature variation on the resolution were tested in the range of 20–30 °C, and the final column temperature was set at 25 °C. Figure 1 showed the typical chromatogram of the simultaneous stereoisomeric separation of hesperidin and narirutin epimers under the optimized chromatographic condition.

### 2.2. Configuration Confirmation

**Figure 2:**
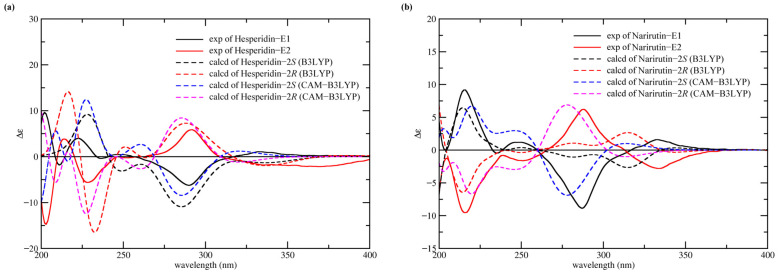
Experimental and calculated ECD spectra of the hesperidin (**a**) and narirutin (**b**) epimers.

In order to confirm the conformation of each epimer, the ECD spectra of them were measured respectively and then compared with the theoretically calculated ECD spectra of the 2*R*- and 2*S*-epimers based on time-dependent density functional theory (TDDFT). According to the previous literature, signs at 280–290 nm (π-π* transition) and at 330–340 nm (n-π* transition) were related to the absolute configuration [18]. As depicted in Figure 2a, the ECD spectrum of hesperidin-E1 (the first eluted epimer) showed the same cotton effect as that of its 2*S*-epimer at 280–290 nm and 330–340 nm. In contrast, the cotton effect in the spectrum of hesperidin-E2 (the second eluted epimer) at the same wavelengths corresponded to that of 2*R*-hesperidin. Similarly, the results in Figure 2b show that the spectrum of narirutin-E1 was consistent with that of its 2*S*-isomer, and the spectrum of narirutin-E2 corresponded to that of its 2*R*-isomer. It is worth noting that the calculated ECD spectra did not exactly match the measured data, and, therefore, we applied two different DFT functionals in the performance of theoretical ECD calculations to obtain a reliable result in the configuration confirmation.

### 2.3. Method Validation

The performance of the developed method was validated as described in Section 3.5. As shown in Table 2, the established method possessed a good linearity, with the coefficient of correlation being higher than 0.9993 at the concentration of the stereoisomer in the range of 0.0055–0.3598 mg/mL.

The corresponding data of the recovery and precision as well as the stability are summarized in Table 3. The relative recovery values varied from 101.1 to 121.9% for each epimer of hesperidin and narirutin. The intra-day and inter-day precision were in the range of 0.79–3.93% and 0.64–3.52%, respectively. The stability test indicated that the sample solution was stable from 0 to 24 h, with RSD values of the content lower than 5.0%, indicating a good stability of all target analytes.

### 2.4. Quantitative Analysis of Samples

**Figure 3:**
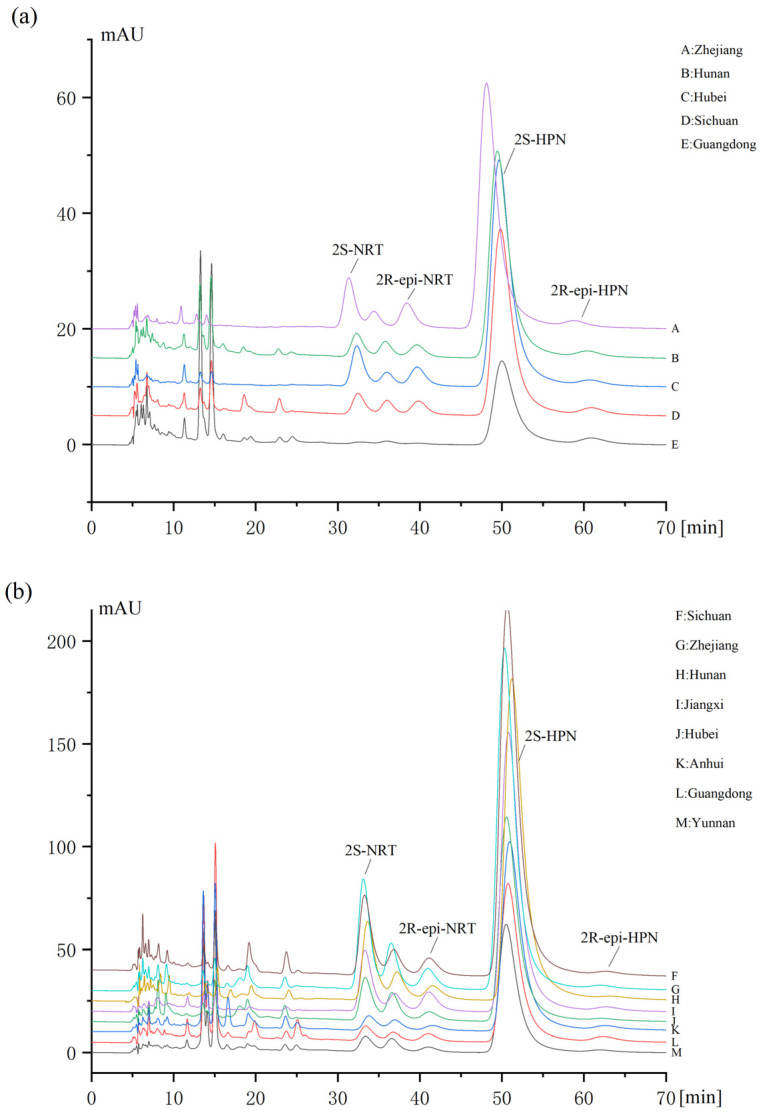
Detection of 2*S*- and 2*R*-epimers of hesperidin and narirutin in Chenpi (**a**) and Qingpi (**b**) herbs of different origins. HPN: hesperidin; NRT: narirutin.

The contents of the individual epimers of hesperidin and narirutin in Chenpi and Qingpi were simultaneously quantified using the developed chiral NP-HPLC method. Figure 3 shows the chromatograms of 2*S*-narirutin, 2*R*-narirutin, 2*S*-hesperidin and 2*R*-hesperidin detected in Chenpi and Qingpi from different producing areas. The content of the 2*S*-epimer as well as the ratio of the 2*S*/2*R* of hesperidin and narirutin are shown in Table 4. For hesperidin, the 2*S*-epimer was predominant in both Chenpi and Qingpi, and the contents were 2.24–6.80% in Chenpi and 4.88–11.3% in Qingpi, respectively. A small amount of 2*R*-hesperidin was present in Chenpi, and almost none was found in Qingpi. In addition, the content of 2*S*-hesperidin varied widely among Qingpi crudes from different production regions, but the ratio of 2*S*/2*R* varied slightly.

Narirutin, which has the same sugar moiety (rutinose) as hesperidin, exhibited different results from the stereoisomeric level. It was found that the ratio of 2*S*/2*R* of narirutin in Chenpi was nearly 1:1. While in Qingpi, the contents of the 2*S*-epimer were several times higher than those of 2*R*-narirutin, which is inconsistent with the previous reports [18]. Based on that, we conceived that there may be a conversion from the 2*S*- to 2*R*-epimer in narirutin during the growth of plants. In addition, the presence of narirutin was not detected in Sample E, which was probably attributed to the different source between Sample E and others. Sample E dried from the ripe peels of *Citrus reticulata* ‘Chachi’ and *C. suhoiensis* Tanaka [22], while other samples were from *Citrus reticulata* Blanco and its cultivated variants. 

### 2.5. Anti-Inflammatory Activities

**Figure 4:**
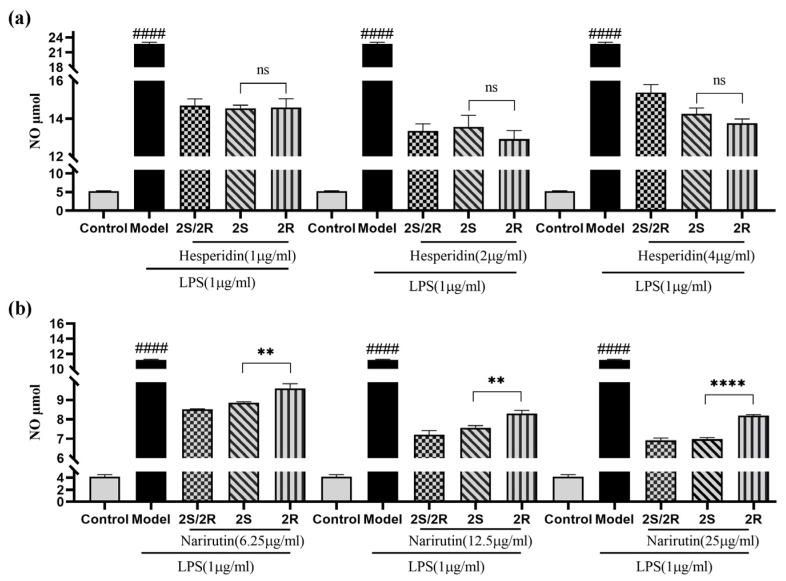
Effects of hesperidin and narirutin epimers on LPS-induced NO production in macrophage cells. (**a**) Effect of hesperidin isomers on LPS-induced NO production in LPS-stimulated RAW 264.7 macrophages. (**b**) Effect of narirutin isomers on LPS-induced NO production in LPS-stimulated RAW 264.7 macrophages. All data are presented as the means ± SEM of three independent experiments. #### *p*< 0.0001 vs. Control group. ** *p* < 0.01 and **** *p* < 0.001 vs. S-epimer group. ^ns^ not statistically significant.

Nitric oxide (NO) acts as one of the crucial signaling molecules in the pathogenesis of inflammation-related diseases. It was reported that hesperidin and narirutin possess anti-inflammatory activity [23,24]. To investigate the difference in the activity between the epimers, all of the isolated epimers were prepared to evaluate their anti-inflammatory, inhibitory effects against NO production in LPS-induced RAW 264.7 macrophage cells. Indomethacin was used as a positive control, and to avoid the effect of the cytotoxicity of these tested epimers, cell viability was assessed by the MTT method first (Figures S2 and S3). Figure 4a shows that 2*R*-hesperidin seemed to have a better inhibitory activity against NO production in LPS-induced RAW cells, while no significant difference was observed between the stereoisomers in our tested concentration range. Regrettably, due to the cytotoxicity of hesperidin, we did not enlarge their concentration for further study. As for narirutin, a significant difference in inhibitory activity between the epimers was observed. Figure 4b shows that 2*S*-narirutin was more effective than 2*R*-form in all tested concentration ranges, but neither was as efficacious as the *R*/*S* mixture, suggesting that the stereo configuration may have some influence on the bioactivity of chiral flavanone to some extent. 

## 3. Materials and Methods

### 3.1. Sample Materials

Crude drugs of Chenpi (Samples A–E), and crude drugs of Qingpi (Samples F–M) of *Citrus reticulata* Blanco were purchased from the main commercial herbal markets and different herbal shops in China. The vouchers have been deposited in Binzhou Medical College (Yantai Campus).

### 3.2. Chemicals and Reagents

HPLC-grade n-hexane (ACS, Wilmington, NC, USA), formic acid (Kermel, Tianjin, China) and isopropanol (≥99.9%, HPLC/ACS-grade) for HPLC analysis were purchased from Energy Chemical Co., Ltd. (Shanghai, China). HPLC-grade methanol was obtained from Concord Technology Co., Ltd. (Tianjin, China). The chiral flavonoid standards of hesperidin and narirutin (mixture of one pair of epimers) were purchased from Shanghai yuanye Bio-Technology Co., Ltd. (Shanghai, China).

### 3.3. Instrumentation and Conditions

Stereoisomeric determination was carried out using an HPLC system (ThermoFisher Scientific, Waltham, MA, USA), which included an UltiMate 3000 pump, an UltiMate 3000 autosampler and an UltiMate 3000 thermo stable column compartment, as well as a diode-array detector. The Chromeleon 7.2 Chromatography Data System (ThermoFisher Scientific) was used for system control and data acquisition. The mobile phase consisted of n-hexane (A) and isopropanol doped with 0.25% formic acid (B) at the proportion of 62:38. Chiral ND (2) column (FLM Scientific Instrument Co., Guangdong, China) packed with amylose tris-3,5-dimethylphenylcarbamate coated on silica (5 μm, 250 × 4.6 mm) was used as the chiral stationary phase (CSP). The flow rate was 0.6 mL/min, and the other parameters were set as follows: a temperature of 25 °C, a detection wavelength of 283 nm and an injection volume of 10 μL.

The isolation of the individual epimer of hesperidin and narirutin was conducted on Shimadzu (SPD-20A) semi-preparative HPLC (Shimadzu, Kyoto, Japan). Standard solutions of hesperidin and narirutin (*R*/*S* mixture) were prepared into 0.4 mg/mL and 10 mg/mL with methanol, respectively, and then 150 μL of the standard solution was injected into HPLC for the isolation of each epimer. A chiral semi-preparative column packed with the same particles in the analysis but differing in size (5 μm, 250 mm × 10 mm) was used for the separation, and an automatic fraction collector was used for the collection of the eluted epimer. The mobile phase consisted of n-hexane (A) and isopropanol (B). For the isolation of hesperidin and narirutin, the proportion of n-hexane and isopropanol was 45:55 and 50:50, respectively. The flow rate was set at 3.0 mL/min.

The eluting fraction was collected several times by an automatic fraction collector, and then the same factions were merged together. After a water bath with evaporation at 80 °C, each single epimer was obtained. However, to obtain the sufficient amounts for the subsequent study on biological activity, the mentioned procedures were repeated multiple times.

### 3.4. Preparation of Sample Solutions

The crude drug was pulverized to obtain the homogeneous fine powder. A total of 1.0 g of the fine powder was accurately weighed and then added into a flask containing 25 mL methanol. The total mass of the powder, methanol and flask was also weighed. After extraction with reflux under heating for 2 h, the mixture was cooled down to room temperature. Then, the total mass was weighed again. The loss of weight was replenished with methanol. After that, 1.0 mL of the extracting solution was taken out and diluted into 5.0 mL with ethyl alcohol to obtain the real sample solution for the determination of each stereoisomer. Subsequently, all of the sample solutions were filtered through a 0.22 μm nylon syringe filter, and 10 μL was injected into the HPLC-UV system for analysis.

### 3.5. Identification of Different Isomers

The individual epimers of hesperidin and narirutin were separately dissolved in methanol with a concentration of 0.1 mg/mL. After filtering through a 0.22 μm nylon syringe filter, 500 μL was infused into the 0.1 cm path length quartz cuvette. The experimental spectra of the collected fraction were recorded between 200 and 400 nm wavelengths with a response of 0.5 s using the Chirascan qCD spectrograph (Applied Photophysics Ltd., Surry, UK).

The theoretical ECD spectra of the 2*R*/2*S* epimer were calculated by the Gaussian 09 software system according to the TDDFT [25]. First, the conformational search was performed using the MMFF94s force field in the conformational search software CONFLEX. Next, those conformations whose energies were not more than 3 kcal/mol higher than the lowest energy were optimized at the B3LYP/6–31G(d) and CAM-B3LYP levels, respectively. They were checked by frequency calculation and resulted in no imaginary frequencies. The ECD of the conformers was then respectively calculated by the TDDFT method at the B3LYP/6–311+G(d,p) and CAM-B3LYP/6–311+G(d,p) levels with the PCM model in a methanol solution. The final calculated ECD curve was generated by summing the calculated ECD of each conformer based on Boltzmann averaging using SpecDis 1.62. The absolute configurations of the compounds were determined by comparing the calculated ECD with the experimental ECD.

### 3.6. Method Validation

The method was validated for linearity, range, precision and accuracy in accordance with the Chinese Pharmacopoeia 2020 edition [26].

The stock solution of hesperidin and narirutin (*R*/*S* mixture) at a concentration of 0.5 mg/mL was prepared by dissolving the standards into methanol. For the commercial hesperidin mixture, it consists of *R*/*S* epimers at a ratio of 36:64; For narirutin, the ratio was 62:38. The stock solution was then diluted stepwise by a mobile phase to obtain the calibration solutions containing each epimer at different concentrations. Six-point calibration curves for 2*S*-narirutin (5.52–44.2 μg/mL), 2*R*-narirutin (9.69–77.6 μg/mL), 2*S*-hesperidin (35.9–359 μg/mL) and 2*R*-hesperidin (5.86–33.0 μg/mL) were constructed to determine each epimer in the real sample. Taking the concentration of each epimer as the *X*-axis and the peak area as the *Y*-axis, the standard curve was drawn. The regression equations of peak areas versus concentrations were determined, and the correlation coefficients (*r*) were calculated and used to assess the linearity. The content of each epimer in six individual crude samples (Sample B) was determined on the same day for intra-day precision assessment and on three consecutive days for evaluating the inter-day precision. Precision was expressed as the RSD (%) of the measured concentrations. The stability test was conducted by storing Sample B at room temperature and then analyzing it at 0, 2, 4, 8, 16 and 24 h, correspondingly. To determine the recovery, the solution of Sample B was prepared in sextuplicate and then added with the reference substance (approximately 100% of the original amount of each epimer in the sample), prepared as described in Section 3.4, and subjected to HPLC for analysis.

### 3.7. Anti-Inflammatory Assay

#### 3.7.1. Cell Viability

The murine macrophage cell line RAW264.7 was cultured in plastic dishes containing Dulbecco’s Modified Eagle Medium (DMEM; Gibco, NY, USA) supplemented with 10% Fetal bovine serum (FBS; Shanghai XP Biomed Ltd., Shanghai, CN) in a CO_2_ incubator (5% CO_2_) at 37 °C. For hesperidin and narirutin, the cytotoxicity of the 2*S*- and 2*R*-epimers as well as their mixtures against RAW 264.7 cells was assessed by a 3-[4,5-dimethylthiazol-2-yl]-2, 5-diphenyl-tetrazolium bromide (MTT) assay. The cells (3.5 × 10^4^ cells/mL) were seeded into a 96-well plate at 37 °C for 24 h. The cells were then treated with the test compounds (2*S*- and 2*R*-epimers and their mixtures) for 24 h or 48 h. Then, the cells were incubated with 100 μL of 0.5 mg/mL MTT for 4 h at 37 °C to test for cell viability. The medium was then removed, and DMSO (150 μL) was added to dissolve all of the formazan crystals. The absorbance of the formazan solution at 490 nm was measured using a microplate reader (Bio Tek Instruments, Inc., Winooski, VT, USA).

#### 3.7.2. Measurement of NO Production

The production of nitric oxide (NO) was assessed by measuring the content of nitrite ion (NO_2_^−^) in the cultured media using a colorimetric method based on the Griess reaction [27]. The cells in 0.5 mL DMEM supplemented with 10% FBS were seeded in a 24-well plate (2.5 × 10^5^ cells/mL). After 18 h, when the cells reached 100% confluence, the old medium was removed and then treated with different concentrations of test compounds and LPS. The plate was kept in a humidified incubator containing 5% CO_2_ at 37 °C for 24 h until the medium was harvested. The nitric oxide tested kit (Beyotime Biotech, Haimen, China) was used for the measurement of NO Production.

## 4. Conclusions

In this study, an efficient method was proposed for the simultaneous stereoisomeric separation and quantification of two major chiral flavanone glycosides in *Citrus reticulata* Blanco. The developed method was then validated in terms of precision and recovery, linearity and stability with satisfactory results. Moreover, each single stereoisomer of hesperidin and narirutin was achieved by using a chiral semi-preparative column, whose stereo configurations were then determined. Subsequently, hesperidin and narirutin with their corresponding C-2 differential isomers in Chenpi and Qingpi from different production regions were analyzed. It was found that hesperidin mainly existed as its 2*S*-form, while narirutin existed as both 2*S*- and 2*R*-epimers in the crude *Citrus reticulata* Blanco. A cell experiment further indicated that the isomers with different stereo configurations displayed different bioactivities, which provided a new insight for the investigation of chiral active ingredients in natural drugs.

## Figures and Tables

**Table 1:** The chemical structures and pKa values of two flavanones.

Analytes	Structure	pKa ^a^
Narirutin	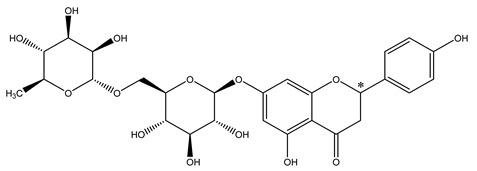	7.18
Hesperidin	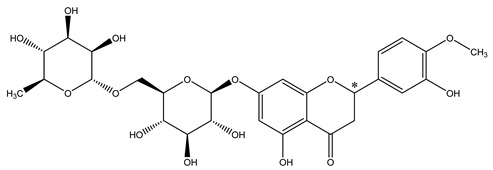	7.15

*: Chiral center. ^a^: predicted pKa values from SciFinder.

**Table 2:** Regression equations, linear range for four analytes.

Analytes	Calibration Curves Equation	*r*	Linear Range (mg/mL)
2*S*-narirutin	Y = 464.4x − 0.7986	0.9993	0.0055–0.0442
2*R*-narirutin	Y = 443.5x − 1.304	0.9994	0.0097–0.0776
2*S*-hesperidin	Y = 456x − 2.850	0.9999	0.0360–0.3598
2*R*-hesperidin	Y = 534.7x − 1.653	0.9998	0.0059–0.0330

**Table 3:** The results of the repeatability, stability, recovery and precision of the four analytes in the samples.

Analytes	Recovery		Precision (RSD%)		Stability (RSD%)
	Mean%	RSD%	Intra-Day	Inter-Day	
	n = 6		n = 6	n = 6	n = 6
2*S*-narirutin	101.1	4.82	1.87	0.94	3.47
2*R*-narirutin	121.9	4.44	1.94	1.34	1.62
2*S*-hesperidin	107.6	5.90	1.68	0.64	0.79
2*R*-hesperidin	114.0	3.96	3.30	3.52	3.93

**Table 4:** Relative ratio of narirutin and hesperidin C-2 diastereomers in *Citrus reticulata* Blanco (peels of Chenpi and Qingpi).

Sample	Origin	2*S*-NRT (%)	2*S*/2*R*	2*S*-HPN (%)	2*S*/2*R*
A	Zhejiang	0.82	57.5:42.5	6.80	96.0:3.96
B	Hunan	0.43	55.2:44.8	5.97	95.7:4.3
C	Hubei	0.67	58.9:41.1	6.12	96.1:3.9
D	Sichuan	0.24	51.2:48.8	3.26	95.0:5.01
E	Guangdong	/	/	2.24	91.3:8.71
F	Sichuan	1.38	75.2:24.8	8.18	98.2:1.85
G	Zhejiang	2.12	81.9:18.1	10.9	98.1:1.91
H	Hunan	1.84	83.0:17.0	11.3	98.4:1.64
I	Jiangxi	1.23	68.8:31.2	8.41	97.5:2.53
J	Hubei	0.83	75.3:24.7	5.92	97.8:2.22
K	Anhui	0.36	65.2:34.8	5.81	96.3:3.66
L	Guangdong	0.35	69.7:30.3	4.92	95.4:4.58
M	Yunnan	0.37	62.8:37.2	4.88	97.0:3.0

Samples A–E: Chenpi; F–M: Qingpi; HPN: hesperidin; NRT: narirutin.

## Data Availability

Most of the data used during the preparation of the manuscript are included in the Results and Discussion sections. However, for any additional details of the procedures and the results’ original raw files, please contact the corresponding authors.

## Supplementary Materials

The following supporting information can be downloaded at: https://www.mdpi.com/article/10.3390/molecules28020873/s1, Figure S1: All chromatograms during the optimization of the separation of hesperidin and narirutin isomers; Figure S2: Effect on cell viability in LPS-stimulated RAW 264.7 macrophages. (a) Effect of LPS on the cell viability of RAW 264.7 macrophages. (b–d) Effect of hesperidin racemates and 2*S*- and 2*R*-isomers on the cell viability of RAW 264.7 macrophages; Figure S3: Effect on cell viability in LPS-stimulated RAW 264.7 macrophages. (a) Effect of Indomethacin on the cell viability of RAW 264.7 macrophages. (b–d) Effect of narirutin racemates and 2*S*- and 2*R*-isomers on the cell viability of RAW 264.7 macrophages; Table S1: The effects of the types of chiral columns and the kinds and proportions of the mobile phase on the separation of hesperidin and narirutin epimers; Table S2: Isolated amounts and recovery rate of the each epimer; Table S3: The relative energies and population of Narirutin-2*R* conformers; Table S4: The relative energies and population of Hesperidin-1*R* conformers.
