# Supplementary material for: Chiral Separation, Configuration Confirmation and Bioactivity Determination of the Stereoisomers of Hesperidin and Narirutin in Citrus reticulata Blanco
Source: Molecules. 2023 Jan 15;28(2):873. doi: 10.3390/molecules28020873 (PMC9867467; doi:10.3390/molecules28020873)
Supplement: Supplementary file 1 [file molecules-28-00873-s001.zip › molecules-2039345-supplementary.pdf]

Supplementary Materials

Figure S1-1

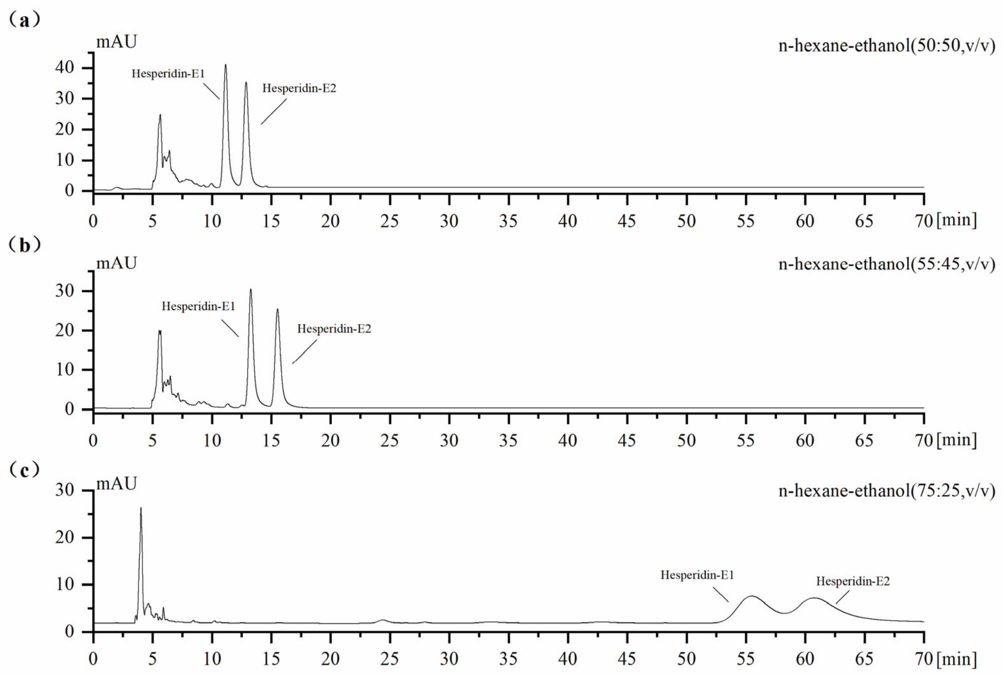

Figure S1-1 Chromatograms of Hesperidin with various mobile phases on Chiral INC column.

Figure S1-2

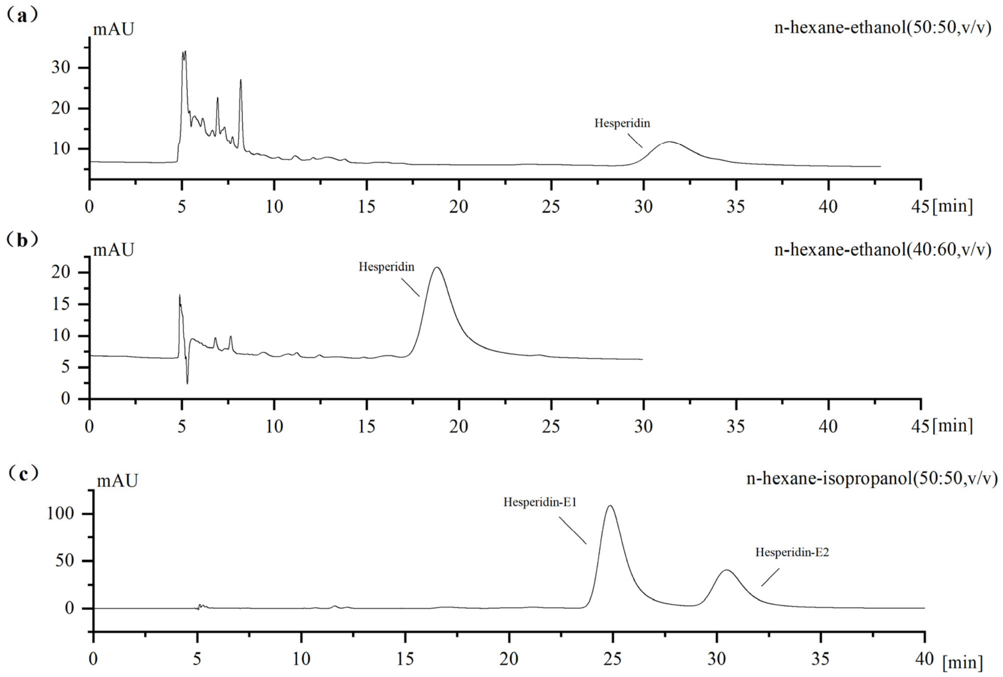

Figure S1-2 Chromatograms of Hesperidin with various mobile phases on Chiral ND column.

Figure S1-3

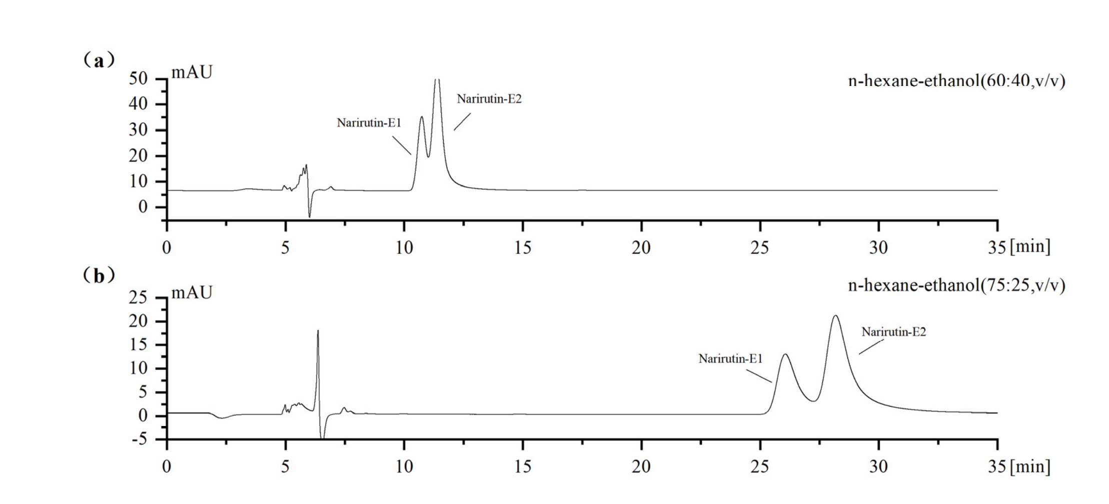

Figure S1-3 Chromatograms of Narirutin with various mobile phases on Chiral INC column.

Figure S1-4

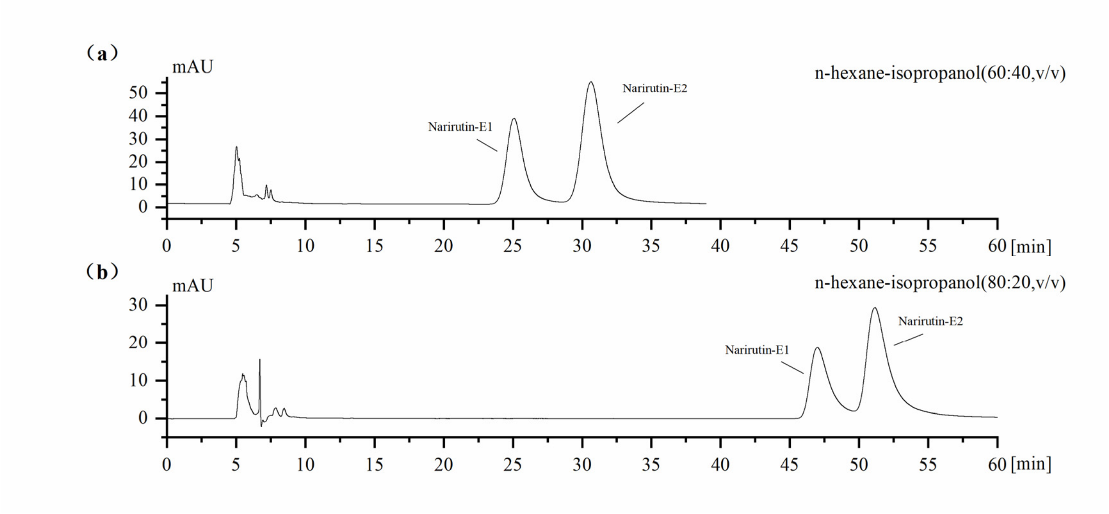

Figure S1-4 Chromatograms of Narirutin with various mobile phases on Chiral ND column.

Figure S1-5

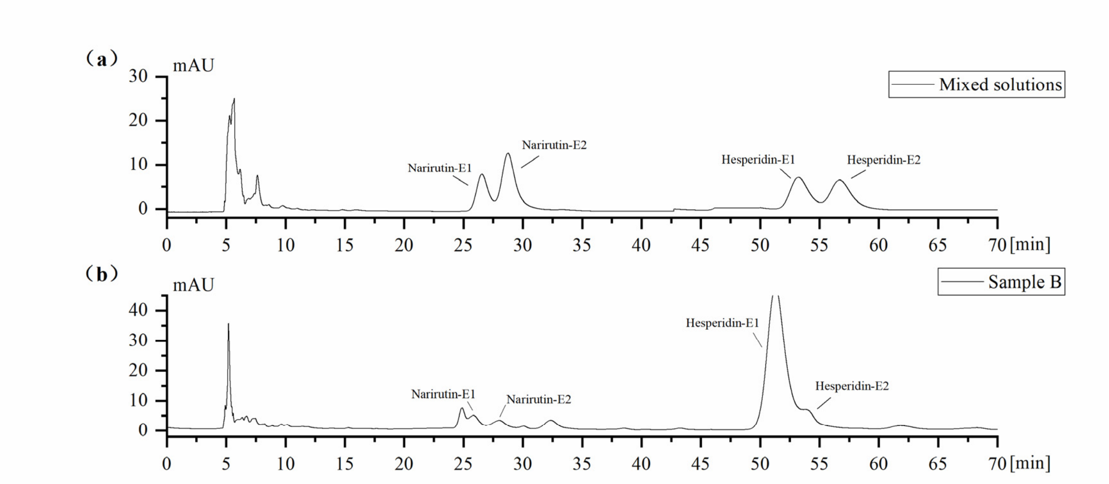

Figure S1-5 Separation chromatograms of the mixed solutions and sample B on Chiral INC column. Mobile phase composition: n-hexane-ethanol (75:25, v/v); column temperature: 25°C; flow rate: 0.6 mL/min.

Figure S1-6

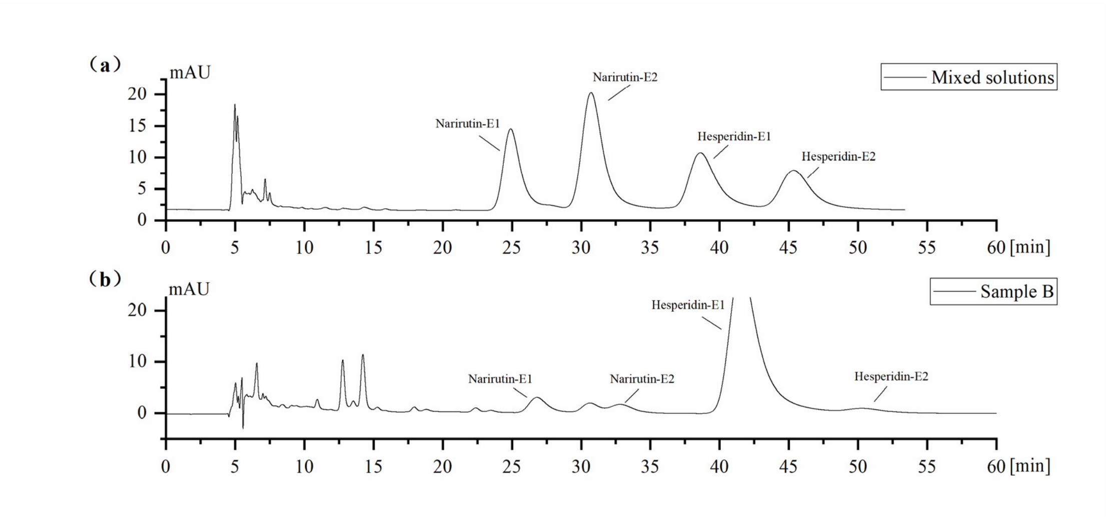

Figure S1-6 Separation chromatograms of the mixed solutions and sample B on Chiral ND column. Mobile phase composition: n-hexane- isopropanol (60:40, v/v); column temperature: 25°C; flow rate: 0.6 mL/min.

Figure S1-7

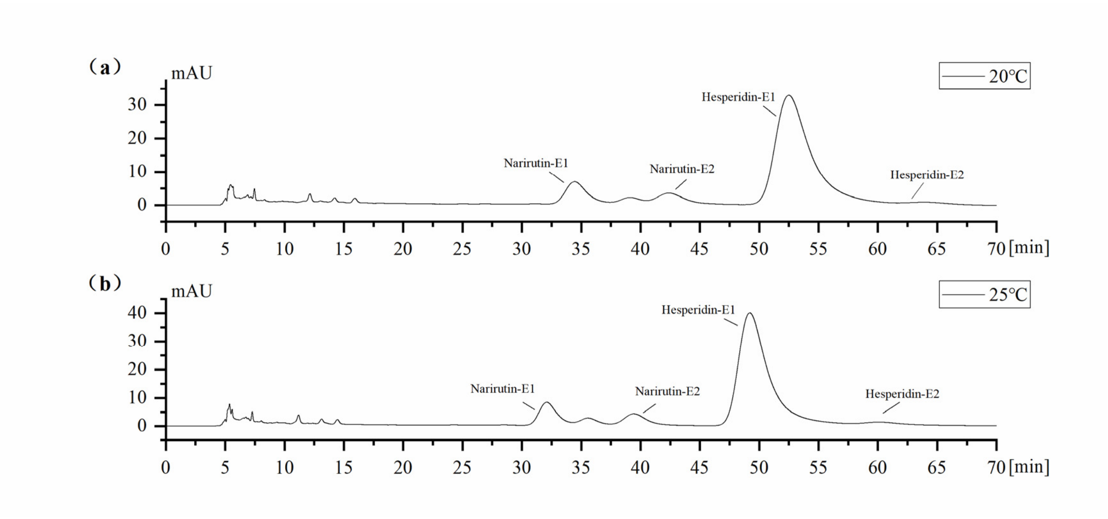

Figure S1-7 Separation chromatograms of sample B on Chiral ND column. Mobile phase composition: n-hexane- isopropanol (62:38, v/v); flow rate: 0.6 mL/min.

Figure S2

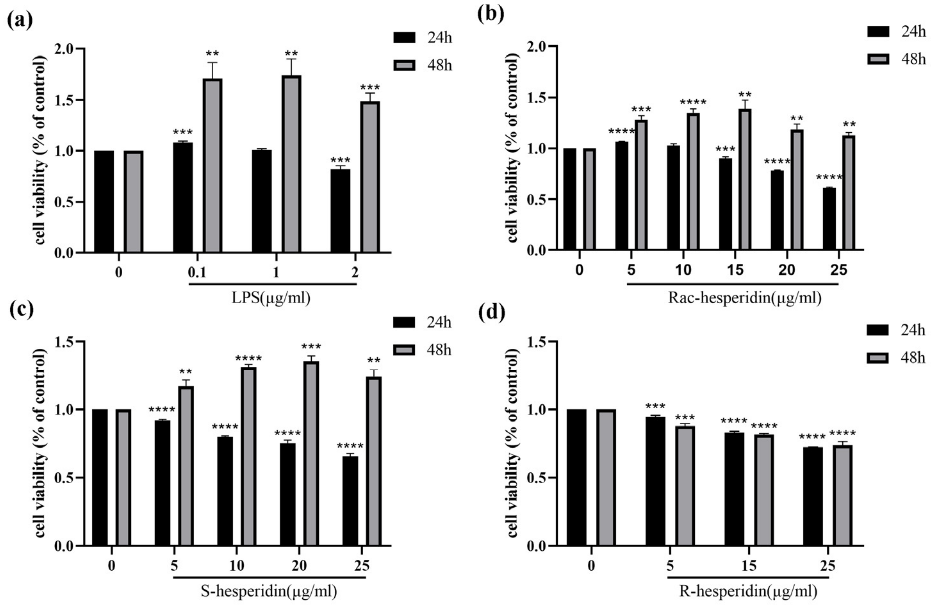

Figure S2 Effect on cell viability in LPS-stimulated RAW 264.7 macrophages. (a) Effect of LPS on the cell viability of RAW 264.7 macrophages. (b-d) Effect of hesperidin racemates and 2*S*- and 2*R*-isomers on cell viability of RAW 264.7 macrophages.

Figure S3

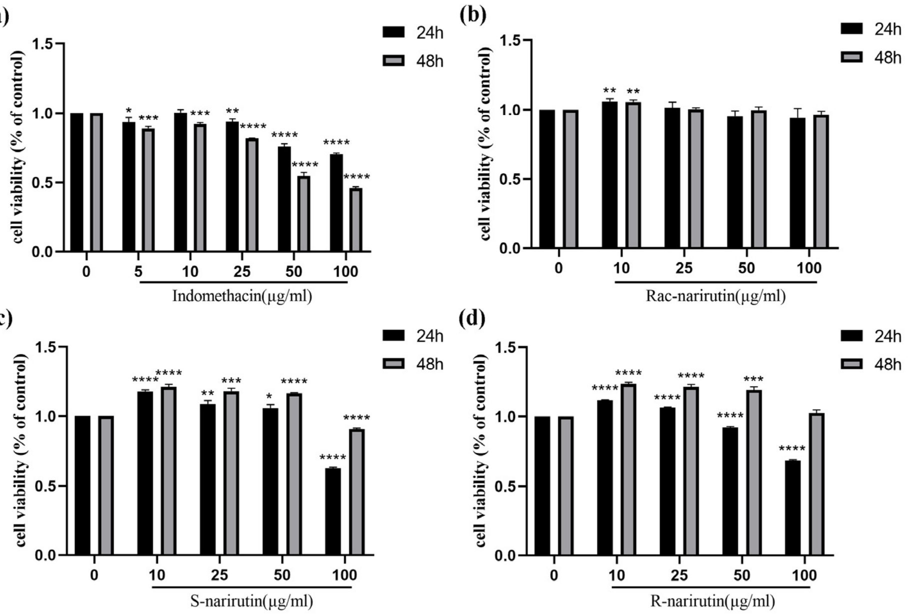

Figure S3 Effect on cell viability in LPS-stimulated RAW 264.7 macrophages. (a) Effect of Indomethacin on the cell viability of RAW 264.7 macrophages. (b-d) Effect of narirutin racemates and 2*S*- and 2*R*-isomers on cell viability of RAW 264.7 macrophages.

Figure S4

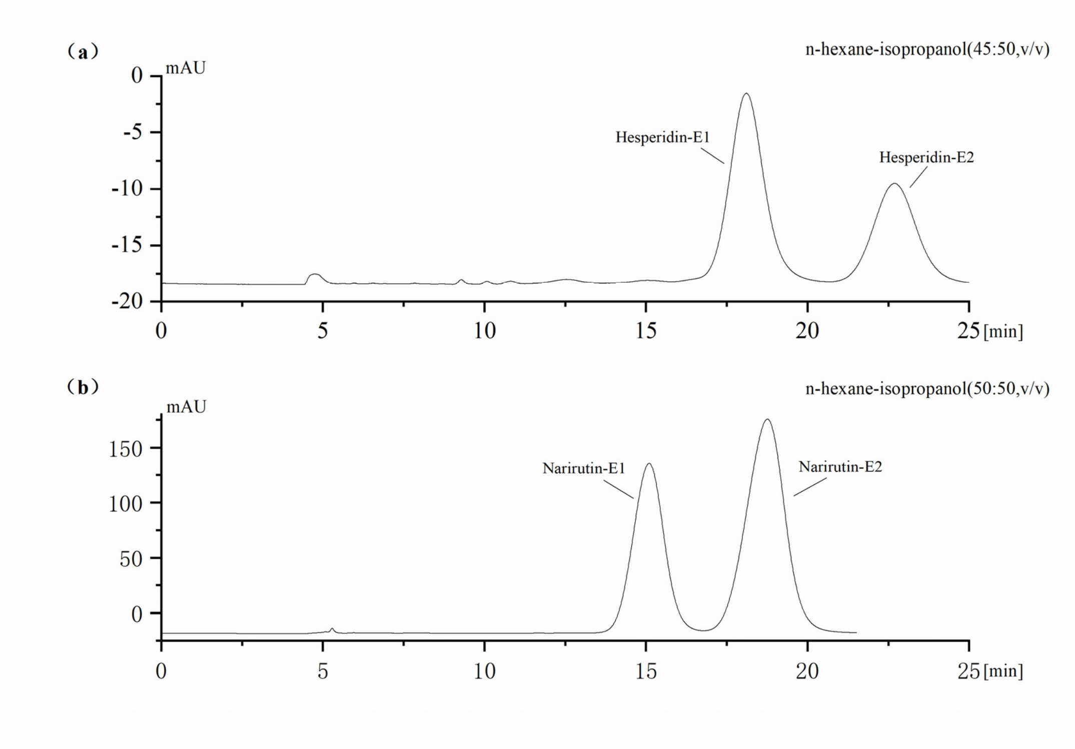

Figure S4 Semi-preparative chromatograms of hesperidin (a) and naringin (b) isomers.

Table S1

The effects of the types of chiral columns, the kinds, and proportions of mobile phase on the separation of hesperidin and narirutin epimers.

Analyte	Column	Modifier	Proportion (%)	k_1	α	Rs
Hesperidin	Chiral INC	ethanol	50	1.2	1.28	2.33
			45	1.7	1.27	2.89
			25	12	1.10	0.94
		isopropanol	30	6.1	1.10	1.12
	Chiral ND	ethanol	60	2.8	/	/
			50	5.2	/	/
		isopropanol	50	4.1	1.28	2.22
			40	6.7	1.20	1.85
			38	8.8	1.24	2.17
Narirutin	Chiral INC	ethanol	40	1.2	1.11	0.89
			25	4.2	1.10	1.31
	Chiral ND	isopropanol	40	4.0	1.27	2.29
			38	5.3	1.26	2.33
			20	7.6	1.10	1.59

k_1: the retention factor of the first eluted enantiomer.

α: the separation factor.

Rs: the resolution.

Table S2

Isolated amounts and recovery rate of the each epimer

Analytes	The starting material(mg)	Component ratio	Recycling amounts(mg)	Recovery rate
2*S*-hesperidin	68.4	0.6101	16	0.3834
2*R*-hesperidin		0.3899	2	0.075
2*S*-narirutin	10.0	0.3912	2.866	0.7356
2*R*-narirutin		0.6088	3.556	0.5864

Table S3

The relative energies and population of Narirutin-2*R* conformers

Conformers of Narirutin-2*R*	Relative Energy (kcal/mol)	Population (%)
1	0.0000	40.8718
2	0.1983	29.2474
3	0.5801	15.3525
4	0.7108	12.3135
5	2.2806	0.8704
6	2.3461	0.7794
7	2.8787	0.3172
8	3.0251	0.2477
9	10.6768	0.0000
10	10.7535	0.0000
11	11.3902	0.0000
12	11.5354	0.0000

Table S4

The relative energies and population of Hesperidin-1*R* conformers

Conformers of Hesperidin-1*R*	Relative Energy (kcal/mol)	Population (%)
1	0.0000	48.3986
2	0.5544	18.9884
3	0.5997	17.5898
4	0.9847	9.1839
5	1.4152	4.4409
6	2.5023	0.7089
7	2.6975	0.5099
8	3.3160	0.1795
9	8.1489	0.0001
10	8.3864	0.0000
11	8.9731	0.0000
12	10.7851	0.0000
